# Plasmid DNA for Therapeutic Applications in Cancer

**DOI:** 10.3390/pharmaceutics14091861

**Published:** 2022-09-03

**Authors:** David Hernán Martínez-Puente, José Juan Pérez-Trujillo, Laura Mireya Zavala-Flores, Aracely García-García, Arnulfo Villanueva-Olivo, Humberto Rodríguez-Rocha, Jesús Valdés, Odila Saucedo-Cárdenas, Roberto Montes de Oca-Luna, María de Jesús Loera-Arias

**Affiliations:** 1 Histology Department, Faculty of Medicine, Universidad Autonoma de Nuevo Leon (UANL), Monterrey 64460, Mexico; 2 Department of Molecular Genetics, Northeast Biomedical Research Center (CIBIN) of IMSS, Nuevo Leon Delegation, Monterrey 64720, Mexico; 3 Departamento de Bioquímica, CINVESTAV-México, Av. IPN 2508, Colonia San Pedro Zacatenco, Mexico City 07360, Mexico

**Keywords:** cancer, DNA vaccination, gene therapy, tumor-specific antigens, apoptosis, plasmids, non-viral gene therapy, tumor-specific promoters, plasmid optimization

## Abstract

Recently, the interest in using nucleic acids for therapeutic applications has been increasing. DNA molecules can be manipulated to express a gene of interest for gene therapy applications or vaccine development. Plasmid DNA can be developed to treat different diseases, such as infections and cancer. In most cancers, the immune system is limited or suppressed, allowing cancer cells to grow. DNA vaccination has demonstrated its capacity to stimulate the immune system to fight against cancer cells. Furthermore, plasmids for cancer gene therapy can direct the expression of proteins with different functions, such as enzymes, toxins, and cytotoxic or proapoptotic proteins, to directly kill cancer cells. The progress and promising results reported in animal models in recent years have led to interesting clinical results. These DNA strategies are expected to be approved for cancer treatment in the near future. This review discusses the main strategies, challenges, and future perspectives of using plasmid DNA for cancer treatment.

## 1. Introduction

According to the World Health Organization (WHO), cancer is a leading cause of death worldwide, with nearly 10 million deaths in 2020 [1]. Different conventional methods and treatments are available for cancer, such as chemotherapy, radiotherapy, and surgical resection. However, if some cancer cells escape these treatments, they can lead to more aggressive tumors [2]; thus, these methods are insufficient. Recently, new therapies have been added to the arsenal to fight cancer with promising results, such as targeted therapy, stem cell therapy, nanoparticles, and active or passive immunotherapy [3,4].

An alternative that has shown promising results is the use of deoxyribonucleic acid (DNA) molecules for gene therapy [5]. Over time, the use of DNA for vaccination against cancer began with the characterization of the first tumor-specific antigen [6]. From there, different strategies have been developed to use this technology in cancer treatment.

The most used DNA-based vectors for cancer gene therapy and DNA vaccination are plasmids, small circular molecules originally obtained from bacteria. Furthermore plasmids, other non-plasmid DNA-based platforms for gene delivery have recently been reported. Some examples of this type of platform are minicircle DNA (the unnecessary plasmid backbone is removed by recombination) [7], MIDGE DNA (minimalistic expression constructs) [8], Doggybone DNA (linear, covalently closed, double-stranded molecules) [9], or linear DNA amplicons produced by polymerase chain reaction (PCR) [10].

Plasmid DNA for gene therapy and DNA vaccination offer several advantages over other nucleic acid platforms, such as being easy to design and manufacture, having a low production cost, and having a high stability for transportation and long-term storage [11].

In this manuscript, we discuss the basics of plasmid design, the use of tumor-specific promoters for gene therapy and tumor-specific antigens for DNA vaccination, the use of fusion proteins to potentiate the antigen immunogenicity, the combination of DNA vaccines with immune checkpoint blockade (ICB), the main in vivo delivery methods, and the principal challenges and future perspectives derived from clinical trials.

## 2. Plasmid Design for Cancer Therapy

**Figure 1:**
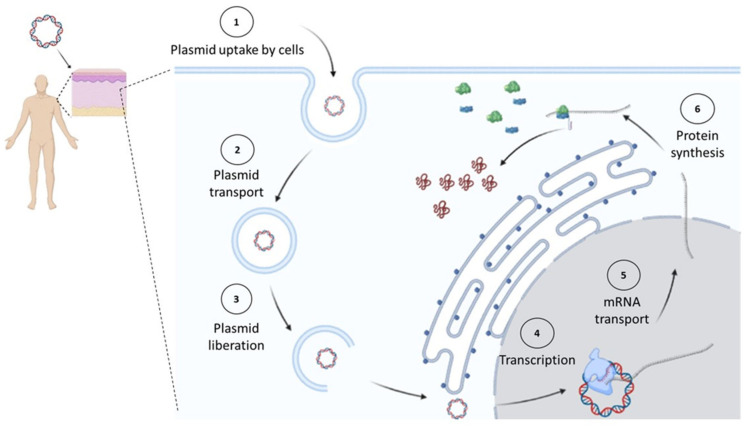
In vivo expression of cancer therapeutic proteins. Once a plasmid enters the cell, it must reach the nucleus, where it will start its transcription by the cell’s machinery. Later, the synthesized messenger RNA (mRNA) will be transported to the cytosol to be decoded by ribosomes into proteins. Figure created in Biorender.com.

Plasmids used for cancer gene therapy or DNA vaccination must contain at least one expression cassette that directs the expression of a protein that will induce the therapeutic effect. After DNA uptake by the cell, it needs to reach the nucleus, where the gene will direct the therapeutic protein expression in the same way the cell produces its own proteins (Figure 1).

**Figure 2:**
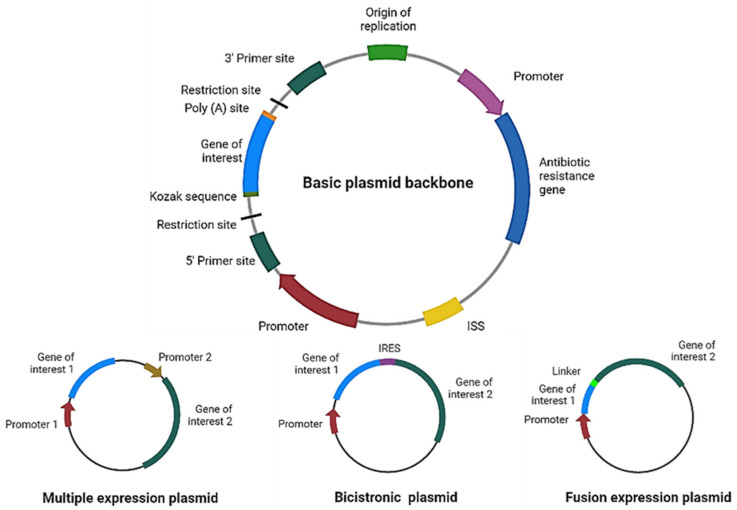
Plasmid design for expression of therapeutic proteins. Schematic representation of the main elements to include in a basic plasmid backbone for cancer therapy and plasmids for expression of multiple proteins. ISS: immunostimulatory sequences; IRES: internal ribosome entry site. Figure created in Biorender.com.

For therapy to be effective, the correct design and optimization of the plasmid are crucial (Figure 2). For example, if more than one gene of interest needs to be expressed using a single plasmid, we can even express them independently (each gene with its own promoter), in a multicistronic system (two or more genes under the control of the same promoter), or as a fusion protein (a linker sequence between both sequences may be added). For the multicistronic system, an internal ribosome entry site (IRES) or a virus-derived T2A sequence must be placed between the different genes [12,13,14,15].

Codon optimization of the gene of interest is highly important, since the richness of guanines and cytosines increases messenger RNA (mRNA) levels [16,17]. Furthermore, the DNA molecule per se may stimulate the immune system through its unmethylated cytosine–phosphate–guanine (CpG) motifs and double-stranded structure [18]. CpG sequences in DNA vaccines have been shown to increase immunogenicity, acting as immunostimulatory sequences (ISS) through recognition by the Toll-like receptor 9 (TLR9) present in antigen-presenting cells (APCs) [19]; however, they may decrease gene expression [20].

**Figure 3:**
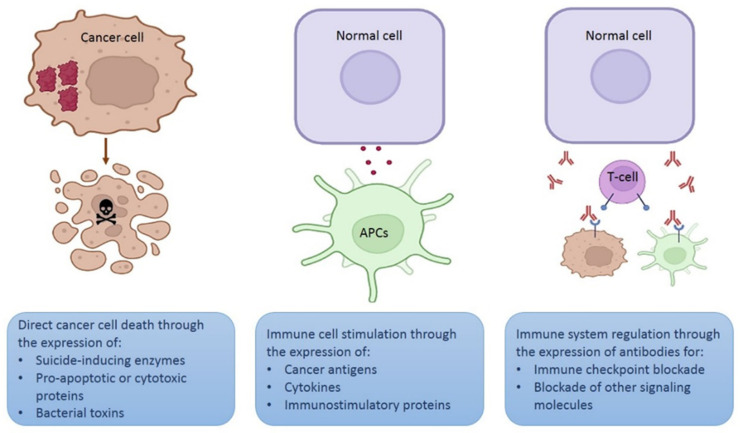
Different strategies using plasmids for therapeutic purposes. Schematic representation of three different strategies involving in vivo expression of therapeutic proteins. Figure created in Biorender.com.

Depending on the strategy intended for the plasmid, the gene of interest may encode a therapeutic protein to kill cancer cells directly, for example, a proapoptotic protein [21], an enzyme that activates a prodrug [22,23], a cytotoxic peptide [24], or a bacterial toxin [25,26]. Plasmids encoding specific small interfering RNA (siRNA) molecules may be used for cancer gene therapy [27,28] (Figure 3). In this case, a tumor-specific promoter can direct the transgene expression in cancer cells [29].

Another option is that the gene of interest encodes an antigen or a cytokine to stimulate immune cells (mainly lymphocytes or APCs) [30,31] to destroy cancer cells. Since a high transgene expression is required for immune stimulation, strong promoters, such as the cytomegalovirus (CMV) promoter, are more suitable for this strategy. Furthermore, expression of the therapeutic protein may be performed by any cell that captures the plasmid. In addition, APC-targeted expression may be achieved using specific promoters [32].

A novel strategy involves using plasmids that encode monoclonal antibodies to block different signaling cascades, such as immune checkpoints or other molecules expressed on the cell surface or secreted in the tumor microenvironment [33].

## 3. Tumor-Specific Promoters for Gene Therapy

As we can find cell- and tissue-specific promoters that regulate the expressions of different genes in normal cells, some promoters also allow for the expression of genes that favor the proliferation of cancer cells. Scientists have taken advantage of the nature of these to allow for the expression of therapeutic genes only in cancer cells. There are promoters functional in cancers of different origin (cancer-specific promoters) but not active in normal cells, and there are specific promoters that are active only in a limited type of cancer cells (tumor-specific promoters) [29]. Herein, we mention some of the most widely used cancer-specific promoters, whose antitumoral effects have been analyzed in vivo using non-viral gene therapy.

The promoter of human telomerase reverse transcriptase (hTERT) has null activity in most somatic cells due to the absence of its methylation, which allows for its binding to the repressor. hTERT is a type of promoter active via methylation in different types of tumor tissues, which allows for the high expression of telomerase, an enzyme responsible for increasing telomeres in the proliferation of cancer cells [34,35]. The therapeutic use of this promoter in cancer therapy has been analyzed in different works. A plasmid that encodes the non-metastatic clone 23, isoform H1 (*nm23-H1)* gene, a metastasis suppressor gene under the control of the hTERT promoter, inhibited tumor growth and distant metastasis when evaluated in a lung cancer xenograft model after intratumoral injection with the vector [36]. In another work, a plasmid that encodes KK-64, a cytotoxic peptide, under the control of hTERT was administered in the form of DNA/liposome complexes to mice previously inoculated with mouse hepatocarcinoma cell line H22, with a reduction in tumor growth observed [37]. A novel version of the hTERT promoter using a VISA (VP16-Gal4-WPRE integrated systemic amplifier) system was reported. In this work, the hTERT-VISA system was used to drive the expression of E1A, an adenoviral transcription factor with anticancer properties. Significant antitumor activity was reported in an ovarian cancer xenograft murine model after intravenous delivery of the plasmid/liposomal nanoparticles [38].

The *BIRC5* gene is active in different cancers but not in normal tissues. It drives the expression of survivin, an apoptosis inhibitor important for cancer development [39]. This promoter has been used in a minicircle system with potential clinical use for prostate cancer diagnosis and treatment [40]. In another work, the survivin promoter was used in combination with hTERT promoter to form a hybrid promoter to increase its strength of expression in transfected cancer cells. This hybrid promoter directed the expression of Herpes simplex virus-1 thymidine kinase (HSVtk) and the mouse granulocyte-macrophage colony-stimulating factor (GM-CSF). These transfected cancer cells were implanted in mice, and tumor growth inhibition was observed [41].

A candidate promoter for breast cancer is Erb-B2 receptor tyrosine kinase 2 (*ERBB2)* gene promoter; however, this is expressed in only 20–25% of tumors [42,43,44], and it is also active in prostate, pancreas, colon, and ovary cancer cells [45,46,47]. The *ERBB2* gene promoter has been used in some works, as in a clinical trial for breast cancer where the patients received intratumoral injection of a plasmid that encodes the *E. coli* cytosine deaminase under the control of the *ERBB2* gene promoter to activate the prodrug fluorocytosine [48]. In another work, a plasmid containing a minimum version of this promoter directing the expression of HSVtk to confer selective cytotoxicity to ganciclovir was constructed and proved in nude mice bearing human breast cancer cells. The administration of ganciclovir in human breast cancer cells transfected with this plasmid reduced tumor growth [49].

Regarding lung cancer, the thyroid transcription factor-1 (TTF-1) promoter is active in small cell lung carcinoma and adenocarcinoma [50,51]. Low constitutive expression is found in healthy lung cells, such as type II alveolar cells [52]. The use of this promoter to drive the expression of miR-7, a powerful tumor suppressor, was reported. This study showed the targeting of transgene expression in the tumor cells via a remote hypodermic injection of a plasmid, downregulating tumor growth in a nude mice model of lung cancer [53].

Prostate-specific antigen (PSA) is regulated by the prostate cancer promoter, which has low constitutive expression in the prostate epithelium [54]; however, high levels are detected in patients with metastatic prostate cancer [55]. It is known that the activity of this promoter can be regulated by DNA-binding proteins [55], and this regulation may be androgen dependent or independent [54,56]. A recently published work reported using liposomes with a vector containing the PSA promoter driving the expression of perforin (a protein that makes pores on the plasma membrane) in cancer cells. After intravenous administration of this therapy, a reduced tumor volume was observed in a xenograft model of prostate cancer [57].

## 4. Tumor-Specific Antigens for DNA Vaccination

To carry out DNA vaccination for cancer therapy, a component of great value is the tumor-specific antigens ((TSAs) antigens expressed only in cancer cells) or tumor-associated antigens ((TAAs) antigens expressed in cancer cells and some normal cells). These are molecules present in tumor cells of different origins, which by synthesizing them as a therapeutic transgene, will help increase the number of epitopes necessary for the activation and stimulation of an antigen-specific immune response [58]. Tumor cells express antigens that, to different extents, can also be found in normal cells, as shown in Table 1.

Different viruses are related to the induction of malignant transformation of cells, such as Epstein–Barr virus (EBV), hepatitis B virus (HBV), hepatitis C virus (HCV), human immunodeficiency virus (HIV), human herpesvirus 8 (HHV-8), human papillomavirus (HPV), human T-lymphotropic virus (HTLV), Merkel cell polyomavirus (MCV) and simian virus 40 (SV40) [93,94]. Some of their viral proteins are considered TSAs because they are expressed exclusively in cancer cells derived from viral-infected cells [95].

Neoantigens are protein derivatives that, during aberrant replication of tumor cells, obtain certain mutations that make them different from the original proteins in a healthy cell. Neoantigens are divided into private (differ among patients) or public (shared among patients) [96].

Some antigens are overexpressed in tumors compared to their expression level in healthy cells. There is a correlation between some tumor-specific promoters and some overexpressed antigens or antigens with aberrant expression. As discussed above, the cause of uncontrolled protein expression lies in the promoter and its regulatory systems, which cause the overexpression of the regulated gene in either a normal or a mutant version (aberrant proteins). These are considered TAAs because they may be expressed in cancer and normal cells [97].

Tumor differentiation antigens are expressed in the tumor cells and normal cells of a specific tissue differentiation germ line [98]. These are also considered TAAs.

Cancer testis antigens are antigens whose normal expression occurs in germ cells. They are not present in adult somatic cells. Their deregulation leads to their expression in somatic cell tumors [99].

Synthetic antigens are artificially modified antigens that enhance immune responses [65]. We can include here multiepitope antigens for personalized cancer therapy [100], mutant versions of viral antigens used to eliminate oncogenic potential [101], or modifications to germline tumor antigens intended to augment immune potency and break immune tolerance [102], among others.

## 5. DNA Vaccines Encoding Fusion Proteins

It has been found that when DNA vaccines are employed alone, there is poor immune stimulation [103]. Different modifications can be designed for DNA vaccines to improve immune responses. One of these is the fusion of the antigen of interest with other antigens or immune-stimulating proteins. The resulting proteins are named fusion proteins or chimeric proteins. Evidence of the use of fusion proteins with promising results is discussed in this section.

### 5.1. Antigen Fusion to Organelle-Targeting Sequences

In 1999, Chen et al. [104] demonstrated the powerful antitumor effect of a DNA vaccine whose therapeutic gene is the result of the fusion of the E7 antigen of HPV-16, linked to the sorting signals of the lysosome-associated membrane protein-1 (LAMP-1) and a signal peptide at its amino-terminal of the tissue plasminogen activator (TPA). The TPA signal peptide is a signal that directs the expression of the therapeutic transgene to the endoplasmic reticulum (ER). This vaccine was implemented in a murine cervical cancer model where a powerful antitumor effect was obtained, mediated by E7-specific cytotoxic T lymphocytes (CTLs) and E7-specific antibodies, controlling hepatic and pulmonary metastasis in comparison with the E7 antigen alone.

Lysosome-targeting by antigen fusion to LAMP1 is still applied in different cancer models. A recent work by Adhikari et al. [105] reports the design of a multi-epitope DNA vaccine using a universal intracellular targeted expression (UNITE) platform, which involves the fusion of antigens to LAMP1 with the aim of improving CD4 and CD8 mediated anti-tumor responses. This strategy generated strong cellular and humoral immune responses and enhanced survival in a murine model of glioblastoma.

Calreticulin, a resident chaperone of the ER, has been used in DNA vaccines fused to antigens for ER targeting through its signal peptide. Cheng et al. [106] reported for the first time that treatment with a DNA vaccine encoding the fusion of E7 to calreticulin elicited an antigen-specific immune response mediated by CD8+ T cells in a murine cancer model.

Our research group has designed several DNA vaccines with enhanced antitumor effects by using E6 and/or E7 antigens from HPV-16 fused to a signal peptide from calreticulin to direct the antigen expression to the ER using the biolistic delivery method in a murine cancer model [107]. In addition, the importance of using a KDEL sequence for antigen retention in ER has been evaluated [108]. Other groups have reported the fusion of cancer antigens with different signal peptides for ER targeting [109,110].

In 2021, we designed a therapeutic transgene under the CMV promoter, which contains the HPV-16 E7 antigen fused to the cyclooxygenase (COX)-2 protein (an ER resident enzyme involved in inflammatory responses). The expression of this construct was directed to the ER by the presence of a signal sequence from COX-2, activating the ER stress response and the unfolded protein response (UPR) induced by protein accumulation in this organelle. In addition, this fusion protein induced antigen degradation by the ER-associated degradation (ERAD) pathway due to the presence of a 19-amino acid COX-2 degradation cassette. The results show the powerful antitumor effect of this fused antigen in murine prophylactic and therapeutic cancer models [111]. Furthermore, when the catalytic domain form COX-2 is deleted (but the signal peptide and ERAD sequence are conserved), the anti-tumor response is similar to the fusion to complete COX-2, demonstrating that the anti-tumor effect is dependent on ER and ERAD targeting [112].

### 5.2. Antigen Fusion to Heat Shock Protein (HSP) 70

HSP70 is a protein that functions as a molecular chaperone [113]. It is also recognized that HSP70 stimulates antitumor responses by transporting tumor-derived immunogenic peptides, stimulating antigen presentation, or being recognized as a natural immunogen when using HSP sequences from different species [114].

Since the end of the 20th century, the fusion of HSP70 with antigens has been used to induce potent antitumor responses [115]. Dickkopf-1 (DKK1) is an antigen that has been used in protein fusions with HSP70. It is an antigen associated with multiple myeloma, which significantly decreases tumor development, prophylactically or therapeutically, allowing for the survival of the murine model in which they were evaluated [116].

Mucin 1, cell surface associated (MUC1) has also been reported as applicable in fusion with HSP70; in this case, the MUC1 protein was modified for secretion. The DNA vaccine was applied in prophylactic and therapeutic murine models of melanoma, inducing the suppression of cell growth of tumor cells expressing MUC1 and increased proliferation of antigen-specific lymphocytes [117].

In another work, a modified version of the E7 antigen from HPV-16 was fused to HSP70 from *Mycobacterium tuberculosis*. When a DNA vaccine was administered, a more significant therapeutic effect against E7-expressing tumor cells in prophylactic and therapeutic assays in mice was observed [118].

Other strategies involving antigen fusion to other HSP proteins, such as HSP60 [119], and antigen fusion to other chaperones [120] are also reported for DNA vaccination, showing promising results.

### 5.3. Antigen Fusion to Cytokines

Cytokines are small proteins secreted by cells for communication and signaling between them. Cytokines have been useful in the investigation of cancer vaccines [121]. Some of the cytokines used are encoded alone [122] or in combination with other cytokines [123] to induce systemic or local antigen-independent immune activation when administered by DNA vaccination [124]. The other strategy is the combination of cytokines with antigens, even using them as independent transcripts [125], or as fusion proteins.

The chemokine macrophage inflammatory protein-3α (MIP-3α), also known as C-C motif chemokine ligand 20 (CCL20), is a cytokine with a strong chemotactic effect on lymphocytes. Recent studies have reported the fusion of MIP-3a to the melanoma glycoprotein 100 (gp100) antigen in the context of a DNA vaccine. Administration by intramuscular electroporation generates a strong antitumor response in a murine melanoma model, causing an increase in CD4+ and CD8+ T lymphocytes, the latter being significantly higher than the control vaccine without chemokines [126].

Biragyn et al. reported the construction of protein and DNA vaccines by fusing interferon-inducible protein 10 (IP-10) and monocyte chemotactic protein 3 (MCP-3) to lymphoma Ig variable regions (sFv). They observed that DNA vaccination with the plasmids encoding the fusion proteins induced more potent protection against a tumor challenge than protein vaccines. In addition, they report that this fusion converted a non-immunogenic antigen into a potent immunogen, inducing a T-cell-mediated antitumor immunity. This group has suggested targeting antigens to APCs for chemokine receptor-mediated uptake as a mechanism responsible for the antitumor effects [127].

Other evidence that reflects the efficacy of using interleukin (IL)-2 with the E7 antigen dates back to 2007. In this work, a group of researchers demonstrated that the fusion of E7 to IL-2 administered by biolistic has a powerful antitumor effect and leads to the strong response of antigen-specific lymphocytes with respect to the use of IL-2 and the antigen alone [128].

### 5.4. Antigen Fusion to Other Immune-Stimulating Sequences

Other workgroups have evaluated the antitumor effects that specific antigens can have when fused to other immune-stimulating sequences. Recent work from Wang et al. showed that the melanoma-associated antigen D4B (MAGED4B) and four-jointed box kinase 1 (FJX1) antigens in a DNA vaccine against head and neck squamous cell carcinoma (HNSCC) caused a powerful antitumor response in murine models. In this work, these antigens were fused to the Dom sequence of the C fragment of tetanus toxin as a stimulant of the activation of CD4+ T lymphocytes and the murine IgH signal peptide that directs its expression to secretion [129].

In the context of modeling cancer with HPV-16, the use of B-cell-activating factor (BAFF) was reported. As its name implies, BAFF is a stimulant of B and T cells, characterized by being a membrane protein secreted after synthesizing by the ER–Golgi system. This protein was fused to the E7 antigen of HPV-16 and was used as a DNA vaccine in a murine model, where an increase in CD8+ T lymphocytes for E7 was observed, which counteracted tumor growth in mice, promoting their survival. In addition, it was observed that the expression of E7 is directed to the ER by BAFF, this being the main factor that potentiates this DNA vaccine [130].

In turn, our research group has reported a DNA vaccine encoding the E7 antigen of HPV-16 fused to the calreticulin signal peptide and to SA-4-1BBL, an oligomer of the ligand that binds to the 4-1BB receptor that works with innate, adaptive pleiotropic effects. We observed the antigen being targeted to the ER by the signal peptide in vitro and a powerful antitumor response in vivo. This response was directed by T lymphocytes specific to the E7 antigen in a murine model of HPV with E7-expressing cells, showing prophylactic and therapeutic efficacy [131].

## 6. DNA Cancer Vaccines in Combination with ICB Therapies

Recently, several checkpoints for the regulation of immune responses have been reported. Different studies in animal models and humans have demonstrated that ICB therapy (mainly using monoclonal antibodies) may improve the antitumor responses of T lymphocytes against cancer cells. Some examples of inhibitory checkpoints are PD-1/PD-L1 and CTLA-4/B7-1/B7-2, among others [132]. Since 2011, the United States Food and Drug Administration (FDA) has approved ICB therapies for different cancers [133,134]. Although some patients treated with ICB therapy show promising results, not all patients respond to it. Therefore, new strategies have emerged in combining ICB therapy with plasmid DNA vaccines encoding TSAs or TAAs, showing that the antitumor effect of gene therapy is potentially higher when used in combination than when used alone [135].

Using a DNA vaccine encoding B16 NY-ESO-1 T cell epitopes (SCIB2) in combination with regulatory T cells (Treg) depletion, anti-CTLA-4 or anti-PD-L1 produced different T cell responses and effects in tumor growth in mice. In particular, it led to a greater emphasis on the combination of SCIB2 with PD-1 since the researchers observed less associated toxicity and complete tumor regression compared to the other combinations [136].

The effect of the combination of a plasmid DNA that encodes either ovalbumin (OVA) or the gp100 antigen adjuvanted with a plasmid that encodes IL-12, combined with anti-CTLA-4 and anti-PD-1 ICB therapy, was analyzed in a B16F10 murine melanoma model. Combined therapy showed strong activation of the antigen-specific immune response and elevated production of antigen-specific antibodies and an increase in intratumoral T CD8+ infiltration [137].

A murine mastocytoma P815 tumor model was used to analyze a therapeutic DNA vaccine encoding the P815A antigen in combination with anti-CTLA-4 and anti-PD-1 ICB therapy. The combined therapies induced a delay in tumor growth and enhanced antigen-specific T cell infiltration in tumors compared to individual therapies [138].

Another study reported the synergy of a DNA vaccine encoding the TERT antigen in combination with an anti-CTLA-4 or anti-PD-1 ICB therapy. They observed that the combination therapy, especially with anti-CTLA-4, induced a better antitumor response than the ICB or DNA vaccine alone. This effect was analyzed in a murine model with TC-1 tumors [139].

In two models of murine colon carcinoma with MC38 and CT26 cells, a DNA vaccine that carries eight neoantigens of the MC36 cell line was combined with an anti-CTLA-4 ICB therapy. In both models, using the combined therapy, an increase in B cells and an increase in neoantigen-specific T lymphocytes were observed, obtaining a significant reduction in tumor size [140].

In 2021, work was carried out using a DNA vaccine containing the vesicular stomatitis virus glycoprotein (VSV-G) as a carrier of foreign T cell tumor epitopes (pTOP) for the activation of the innate and epitope-specific immune response. The treatment was administered by intramuscular injection followed by electroporation in combination with anti-PD-L1 and anti-CTLA-4 ICB therapy, manifesting a potent antitumor response that increased the survival of mice in different tumor models [141].

E6 and E7 antigens from HPV 16 and 18 were used in a DNA vaccine in combination with a vaccinia boost and anti-PD-1 ICB therapy in mice with TC-1 tumors. In this report, the DNA vaccine encodes the antigens as a fusion protein with a 3′ signal sequence and a 5′ sequence encoding the HSP70 of *Mycobacterium tuberculosis*. The viral antigens E6 and E7 are oncoproteins; thus, point mutations were included to eliminate the oncogenic potential. They referred to this mutated form of the antigens as detox. The resulting plasmid is named pNGVL4a-Sig/E7(detox)/HSP70, or pBI-1. The vaccinia virus expresses the E6/E7 fusion protein and has been tested in several clinical trials, where it was well tolerated but with poor clinical benefit. With this strategy, a good safety profile and therapeutic efficacy were found, alone or in conjunction with the vaccinia boost, with or without the ICB therapy in mice [101].

A DNA vaccine for glioblastoma was recently reported that expresses the VSV-G with the glioblastoma antigen tyrosinase-related protein 2 (TRP2) epitope sequence TRP2_180–188_ inserted in permissive sites. ICBs such as anti-PD-1 and anti-CTLA-4 accompanied this strategy. It was observed that although the combination of DNA vaccine and ICB therapy did not induce a significantly different survival rate in the treated mice, an increase in effector T cells to Treg ratio was observed, as well as an increase in the release of interferon (IFN)-γ by CD8+ T lymphocytes that infiltrated into the brain after the administration of the combined therapy. This effect was analyzed in mice challenged with GL261 cells [142].

## 7. Antibody Production by DNA Immunization

Recently, DNA-encoded monoclonal antibodies (DMAb) have emerged as an elegant strategy to combat viral infections [143], and later, its capacity for cancer treatment application was demonstrated [144]. They consist of synthetic plasmids that direct monoclonal antibody expression in vivo to overcome the limitations of traditional monoclonal antibodies. The main advantages of DMAb are its rapid development and simple manufacturing processes [33].

In 2016, Kim et al. [144] reported that treatment of mice bearing the receptor tyrosine-protein kinase (erbB-2)-positive human breast carcinoma cell line BT474 with a plasmid encoding an anti-erbB-2 DMAb resulted in a sustained antibody expression and an antitumor efficacy similar to four doses of intravenously injected Herceptin antibody.

This strategy has also been reported for prostate cancer using a plasmid that encodes a DMAb directed against the prostate-specific membrane antigen (PSMA) [145]. In this work, the authors observed an in vivo controlled tumor growth and significant survival in mice vaccinated with this plasmid. This antitumor effect may be mediated by antibody-dependent cellular cytotoxicity through natural killer (NK) cells.

Duperret et al. [146] reported the construction of a synthetic plasmid encoding an anti-CTLA-4 monoclonal antibody. They reported that a single dose induces the expression of this antibody for several months. In addition, they observed that treatment with this DMAb induced tumor regression in Sa1N and CT26 tumor models in mice.

In recent work, Perales-Puchalt et al. [147] reported using synthetic DNA that encodes bispecific T engagers (BiTEs, a fusion protein that combines the specificity of mAbs with the cytotoxic potential of T cells). This DNA-encoded Bite (DBiTE) was directed against erbB-2. Its in vivo expression lasted approximately four months with a single dose. Treatment with this kind of DMAb resulted in high T cell cytotoxicity against erbB-2-positive tumor cells and delayed cancer progression in mice.

## 8. Delivery Methods for Plasmids in Cancer Therapeutics

The plasmids used for gene therapy are usually administered directly into the tumor site to target cancer cells. Furthermore, plasmids used for vaccination are usually administered by mucosal delivery (where the presence of APCs improves vaccination efficiency) or by intramuscular, intradermal, or intratumoral injections to target either somatic cells, cancer cells, or immune cells for antigen production [148].

**Figure 4:**
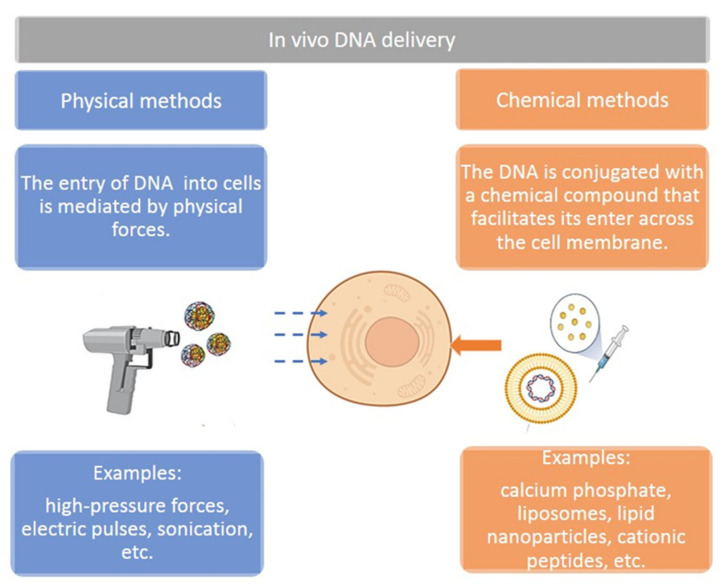
Different strategies using plasmids for therapeutic purposes. DNA delivery methods can be divided into physical- and chemical-mediated methods. Figure created in Biorender.com.

The simplest form of administration of plasmids is the injection of naked DNA. Due to their electrostatic characteristics, such as their negative charge and size, plasmids are often administered with other delivery methods to improve cell entry (Figure 4). This section reviews some of the most commonly used delivery methods for plasmid DNA-based cancer therapies analyzed in in vivo experiments.

### 8.1. Naked DNA Injection

Wolff et al. reported the direct injection of naked DNA for the first time in 1990 [149]. In this work, they injected plasmids encoding different reporter genes in the skeletal muscle of live mice. They demonstrated that transgenes were expressed within the muscle and that expression was present for at least two months.

There is more evidence of the safety of the administration of naked DNA. In 2000, the results of a phase I/II clinical trial for prostate cancer were published, wherein the safety and immunity of a naked DNA vaccine encoding PSMA or CD86 in separate expression vectors or a combined plasmid (PSMA/CD86) were reported. The effects of these plasmids were compared with the use of an adenoviral vector encoding PSMA. Only 50% of patients with naked DNA administration with PSMA and CD86 showed signs of immunization (evidenced by a delayed-type hypersensitivity reaction after treatment). In total, 67% of patients immunized with the PSMA plasmid and recombinant GM–CSF showed immunity, while all patients vaccinated with PSMA/CD86 plasmid and GM–CSF became immunized. Finally, all patients who received the PSMA adenoviral vector and the PSMA plasmid were successfully immunized. No short- or long-term side effects were reported following immunizations [150].

To increase the immunogenicity of naked DNA vaccines against cancer, a working group has proposed the administration of these vaccines in peripheral lymph nodes, where they reported 100- to 1000-fold enhanced immunogenicity, inducing a strong cellular immune response in a murine cancer model. This strategy is promising for improving vaccination immunogenicity in humans [151].

Wu et al. demonstrated in a murine model that the application of naked DNA via systemic administration in the inferior vena cava targets the proximal tubules of the kidneys significantly compared to other organs, such as the lung, liver, and spleen, demonstrating the expression of the β-galactosidase reporter gene in the cell cytoplasm after 30 min of its application, the expression of which was then prolonged for 35 days, without any secondary effect. They propose using this administration route for naked DNA therapy against kidney carcinoma and other kidney diseases [152].

### 8.2. Electroporation

Due to cell membrane impermeability preventing the introduction of genetic material, electroporation (also called electropermeabilization or gene electrotransfer) was developed. Electroporation involves the use of electrical pulses that allow for the formation of small pores in the membrane, through which the plasmids have the opportunity to enter the interior of the cell, with the stimulation of the immune system per se [153]. This technique was developed by Neumann et al. [154] and has demonstrated to be one of the most effective methods for DNA delivery [155].

One of the studies that support the stimulation of the immune system by electroporation is that of Sales et al. This group reported that electroporation stimulates the local migration of antigen-presenting cells, thus allowing for a greater antitumor response in conjunction with a DNA vaccine expressing the fusion of the E7 antigen to the HSV-1 gD protein in an HPV cancer model [156].

Recently, Paolini et al. reported the delivery of plasmids encoding an antibody in single-chain format (scFv) against the HPV-16 E6 and E7 proteins in three different murine preclinical models [157]. They demonstrated the efficient antitumor response induced by scFv delivered as intrabodies by electroporation, with the induction of a delayed tumor progression and large apoptotic areas in tumors.

In 2020, Jacobs et al. compared the antitumor effect of intramuscular and intratumoral electrotransfer of plasmids encoding anti-PD1 and anti-CTLA-4 antibodies in a murine cancer model. They observed a similar antitumor effect between both delivery sites, suggesting the tumor as an appealing delivery site for DNA-based mAb therapies [158].

IL-12 is an interleukin with a proinflammatory action that stimulates CD3+ lymphocytes. IL-12 has been used for several years through DNA vaccination in combination with other immunogens to stimulate the immune system in viral diseases [159] and cancer [160]. Recently, Jacobs et al. reported the intratumoral DNA electroporation in mice with plasmids that encodes IL-12, anti-PD1 and anti-CTLA-4 antibodies [161]. This triple-combination therapy induced CD8+ T cell infiltration in electroporated tumors and a significant anti-tumor response.

In 2022, a working group highlighted the use of DNA vaccines that encode IL-12 and the plasmid that encodes anti-CD3, an intratumoral T-lymphocyte stimulant [162]. They showed their effectiveness through intratumoral electroporation, improving the proliferation of T-lymphocytes and their cytotoxic function, in addition to the production of cytokines.

Intratumoral electroporation with a plasmid that encodes IL-12 in combination with a plasmid that encodes IL-2 has been reported in a murine model of melanoma with B16.F10 cells. In this work, a significant tumor growth delay and regression was observed, with recruitment of CD4+ and CD8+ cells [163].

Several clinical trials are using intramuscular [164] and intratumoral electroporation [165,166,167] for delivery of plasmids encoding IL-12 in combination with TSAs and other immunomodulatory strategies with promising results.

### 8.3. Biolistic

Biolistic is an alternative technique proposed to make gene delivery more efficient, using gold particles that can measure from 1 to 4 µm covered in therapeutic DNA. These are applied through cartridges and a gene gun device that allows for the release of particles at high speed, utilizing helium (a noble gas) at low pressure (200–300 psi) [168]. This technique has been used in plant and animal cells [169].

It has also been observed that when using 40 nm particles, there is an efficient expression of the transgenes of interest with a conventional size; however, the use of nanoparticles allows small cells to be transfected compared to microparticles while decreasing tissue damage [170].

In cancer research, gold particles are covered with therapeutic genes of different kinds, ranging from adjuvants (to stimulate the immune system), such as tumor-specific antigens, to the use of proapoptotic genes to combat this disease. In 1995, treatment of the IFN-γ and IFN-α genes in a murine antitumor model with biolistic led to a significant reduction in tumor growth compared to a control group of mice [171].

In 2009, a study reported the antitumor effect of a naked DNA vaccine encoding calreticulin fused to the E7 antigen by biolistic, comparing gold particles coated with a plasmid (the conventional biolistic technique) and the use of a noncarrier DNA vaccine without any particle coating. This strategy was applied in a murine model of cervical cancer. This work showed an increase in the number of CD8+ T lymphocytes against E7 in mice treated with noncarrier naked DNA, complemented by activation in the production of neutralizing antibodies against E7, and thus an effective antitumor effect. In addition, it was observed that the mice did not have skin burns following the application of noncarrier naked DNA compared with the conventional technique. The use of noncarrier naked DNA delivery by biolistic has the advantage of reducing the costs of the vaccine, as it avoids using gold particles [172].

### 8.4. DNA–Liposome Complexes and Lipid Nanoparticles

Liposomal complexes were developed to facilitate the delivery of DNA to cells since they are composed of phospholipids (similar to the membrane) or have cationic charges increasing the delivery efficiency of the genetic material. Conversely, lipid nanoparticles (LNPs) are sphere-shaped nanovesicles composed of ionizable cationic lipids that permit the encapsulation of nucleic acids in their internal aqueous phase. LNPs have a high encapsulation efficiency and stability, enhanced cellular uptake, and reduced toxicity [173].

One of the first tests in a melanoma model showed high expression of chloramphenicol acetyltransferase (CAT), a reporter gene, by injecting free DNA intratumorally, compared to injecting DNA in the company of cationic liposomes, lipofectamine, and DC-chol/DOPE. This test also reported more efficient expression of genes using the CMV promoter rather than the SV40 or T7 promoters [174].

A comparative study of the delivery efficiency of a plasmid encoding CAT was carried out between complexes of the cationic liposomes that carry DNA aggregates against naked DNA in a murine melanoma model. Using labeling with 3H-thymidine [3H], they detected tumor-associated DNA and liposomal complexes with [14C]-dioleoylphosphatidylethanolamine 24 h after administration. With these data, they observed highly variable expression, a higher transfection rate in small tumors, and efficient liposomal–DNA complex binding to the tumors [175].

To streamline the delivery of genetic material through liposomes, working groups have created neutral or positively charged liposomal complexes containing a folic acid–cysteine–polyethyleneglycol–phosphatidylethanolamine (FA–Cys–PEG–PE) conjugate. These molecules were tested in in vitro and in vivo models with the intraperitoneal application of L1210A cells, corresponding to mouse lymphocytic leukemia, obtaining an optimal delivery range in vitro and in vivo only for the cationic liposome complex with FA–Cys–PEG–PE. This observation was detected through luciferase expression as a reporter transgene, observing a dose-dependent inhibition of the concentration of FA–Cys–PEG–PE. The results show the efficiency of gene therapy delivery with a cationic liposome complex that presents a specific ligand for the folate receptors in cancer [176].

Further evidence of the suitability of using liposomal complexes as DNA carriers is found in the liposomal formulation (extruded DOTAP:cholesterol (DOTAP:Chol)–DNA complex), a cationic complex, which demonstrated that there was a large difference in the expression levels of the luciferase transgene between the in vitro and in vivo models in mouse and human lung tumor cells versus healthy cells. With the use of the liposomal complexes, a high activity of phagocytosis of the complexes in tumor cells was observed [177].

Currently, in cancer research, cationic liposomes are used as vectors for the delivery of therapeutic transgenes [178], even participating in the cytotoxicity of tumor cells. To demonstrate this, Cong et al. made a reporter gene DNA complex with a cationic liposome formed by cholesterol, DOTAP, and DSPE-mPEG2000. The results showed an increase in tumor cell death, promoting the activation of dendritic cells and inhibiting tumor growth and metastasis [179].

Another strategy involving DNA administration in ternary complexes to target dendritic cell uptake yielded promising results. In a model of melanoma, the effect of the pulmonary administration of naked plasmid DNA pUb-M (encoding ubiquitinated murine melanoma gp100 and TRP2 peptide epitopes) or a ternary complex (composed of pUb-M plus dendrigraft poly-L-lysine (DGL), and γ-polyglutamic acid [γ-PGA]) was compared. The administration was by inhalation in mice. The results show the expression of the transgenes of interest in areas with a high concentration of alveolar macrophages. In addition, a significant increase in the inflammatory cytokines of tumor necrosis factor (TNF)-α, interferon (IFN)-γ, and IL-6 was observed. Additionally, significant inhibition of the metastasis of B16-F10 cells, a murine melanoma cell line, was observed with better antitumor effects when using the ternary complex versus the controls [180].

There are some reports using LNPs for DNA vaccine delivery in other murine cancer models. In 2021, Moku et al. report the effect of LNPs functionalized with the mannose-mimicking shikimoyl- and quinoyl-groups for in vivo targeting the mannose receptor of dendritic cells [181]. The subcutaneous administration of LNPs carrying a plasmid encoding the antigen MART1 delayed melanoma growth significantly and improved the survival of mice in a therapeutic assay.

In another work, Liu et al. report the use of lipid-protamine-DNA nanoparticles to drive the expression of “trap” a C-C motif chemokine ligand 2 (CCL2)-binding antibody [182]. CCL2 is a key regulator secreted by tumor-associated adipocytes that induce an immunosuppressive microenvironment. They reported that treatment with LNPs to drive the local and transient expression of trap protein by cancer cells successfully remodels the immunosuppressive tumor microenvironment in a triple negative cancer murine model.

### 8.5. Other Nanoparticle Systems

Recently, other nanoparticle (NP) systems have been increasingly applied in DNA vaccines, as evidence shows that their implementation with plasmids improves delivery systems in cells. Here, we list some works reported in the literature employing different NP systems for DNA vaccination in animal models.

In this context, Sun et al. demonstrated how a DNA vaccine encoding OVA antigen, conjugated with calcium phosphate NPs functionalized with mannose and bisphosphonate, improves the efficiency of vaccine administration, targeting the antigen-presenting cells through C-type lectin receptors (CLRs) and triggering a specific antibody response against the antigen. This effect was analyzed in a murine tumor model with OVA-expressing E.G7 tumor cells [183].

Another strategy is the use of peptide-based delivery systems. RALA is a self-assembled peptide-based cationic nanostructure composed of 30 amino acids developed in 2014, which can deliver nucleic acids and other anionic molecules to the cells crossing the cell membrane with low toxicity [184]. In a prostate cancer model, a strategy has been reported involving a DNA vaccine encoding prostate stem cell antigen (PSCA), delivered via a patch of soluble silicon microneedles containing cationic RALA/pDNA NPs, generating a strong immune response in the tumor [185].

The use of NPs has also been analyzed in NP-coated bacteria. In 2015, a study was carried out wherein a DNA vaccine encoding autologous vascular endothelial growth factor receptor 2 (VEGFR2) was delivered utilizing *Salmonella* coated with cationic polymers, thus evading cellular phagosomes and increasing its dissemination via the blood after oral administration. This delivery method, when orally administered, caused efficient T cell activation, cytokine production, angiogenesis suppression, and tumor necrosis [186].

## 9. Clinical Trials Using DNA Vaccines

In clinical trials, DNA vaccination was safe and well tolerated, with no important adverse effects reported. One of the first concerns about using DNA vaccines is the risk of integration into the human genome, although it has been demonstrated that this risk is low [187]. The FDA guidance for DNA vaccines is that the plasmid integration rate would be substantially lower than the spontaneous mutation rate [188].

Another important challenge in DNA-based therapies is to increase DNA cell uptake. As shown in Table 2, human intramuscular injection followed by electroporation is one of the most efficient delivery methods [189]. Recently, in clinical trials, intratumoral electroporation has been demonstrated to induce tumor regression at distant sites [190], mainly for melanoma and other skin cancers where tumors are accessible for this treatment. However, it has been reported that in mice, intramuscular DNA injection followed by electroporation augments the chances of plasmid integration into host genomic DNA [191]; hence, there is a need for the development of safer and more efficient delivery methods.

When plasmid DNA-based strategies are translated to clinical trials, different results have been reported ranging from non-significant anti-tumor responses to effective therapeutic effects with the induction of antigen-specific CD8+ T cells and tumor regression. One of the main challenges in DNA vaccination is the induction of a potent immune stimulation. Different strategies have been employed to overcome this, such as consecutive vaccine strategies (known as prime-boost immunization) or the administration of DNA vaccination in combination with ICB therapy, other monoclonal antibodies, immunostimulatory molecules, adjuvants, or drugs, among others [192]. Most of the clinical trials with DNA vaccines remain in Phase I–II.

**Table 2:** Clinical trials using DNA vaccines for cancer treatment.

Phase	Type of Cancer	Site of Administration and Delivery Method	Description of Intervention and Key Results	Trial/ Status/ Reference
I	Stage III–IV or Recurrent Ovarian Cancer	Intradermal injection	Intervention: pUMVC3-hIGFBP-polyepitope DNA vaccine encoding Insulin-Like Growth Factor Binding Protein-2 (IGFBP-2) mixed with rhuGM-CSF monthly for three months. Key results: Stimulates the production of type 1 T lymphocytes without evidence of regulatory responses	NCT01322802/ Completed/ [193]
II	Non-metastasic castration-sensitive prostate cancer (CSPC)	Intradermal injection	Intervention: pTVG-HP DNA vaccine encoding PAP with rhGM-CSF. Key results: No overall increase in 2-year metastasis-free survival (MFS).	NCT01341652/ Completed/ [194]
II	Metastatic castration-resistant prostate cancer (CRPC)	Intradermal injection	Intervention: sipuleucel-T with or without pTVG-HP DNA vaccine encoding PAP Key results: The combination of sipuleucel-T with pTVG-HP can increase the diversity of the cellular and humoral immune response.	NCT01706458/ Completed/ [195]
II	Metastasic CRPC	Intradermal injection	Intervention: pTVG-HP is a plasmid encoding PAP, with Pembrolizumab, a (PD-1)-blocking antibody No study results are available	NCT04090528/ Recruiting/ [196]
I	Head and Neck Cancer	Intramuscular injection and electroporation	Intervention: pNGVL-4a-CRT/E7 (detox) DNA vaccine encoding calreticulin and HPV-16 E7 antigen with cyclophosphamide No study results are available	NCT01493154/ Terminated/ [197]
I	Nine types of cancer	Intramuscular injection and electroporation	Intervention: INO-1400 or INO-1401 Plasmid encoding hTERT variants, with or with-out plasmid encoding IL-12 Key results: Survival of patients with pancreatic cancer, tolerance, enhanced CD8^+^ response	NCT02960594/ Completed/ [164]
I	Prostate cancer	Intramuscular injection and electroporation	Intervention: INO-5150 encoding PSA and PSMA with and without INO-9012 encoding IL-12 Key results: Dampening percentage rise in PSA and increased PSA Doubling Time (PSADT) in patients.	NCT02514213/ Completed/ [198]
IB	Breast Cancer	Injection and electroporation	Intervention: Mammaglobin-A DNA vaccine No study results are available	NCT02204098/ Recruiting/ [199]
I, II	Cervical intraepithelilal neoplasia (CIN) 2/3	Intramuscular injection	Intervention: VB10.16 vaccine (HPV-16 E7/E6 protein linked to human chemokine MIP-1α) Key results: Tolerance and promising immunogenicity results dependent on specific T lymphocytes	NCT02529930/ Completed/ [200]
I, IIA	Cervical Cancer	Intramuscular injection and electroporation	Intervention: INO-3112 DNA vaccine (VGX-3100 encoding for modified HPV-16 and HPV-18, E6 and E7 antigens, and INO-9012 encoding IL-12) No study results are available	NCT02172911/ Completed/ [201]
I, IIA	Head and Neck Cancer	Intramuscular injection and electroporation	Intervention: MEDI0457 (DNA immunotherapy targeting HPV16/18 E6/E7 with IL-12 encoding plasmids) in combination with Durvalumab for PD-1/PD-L1 blockade Key results: Durable antigen-specific peripheral and tumor immune responses.	NCT03162224/ Completed/ [202]
II	CIN 3	Intramuscular injection and electroporation	Intervention: GX-188E is a DNA vaccine encoding HPV-16 and HPV-18 E6/E7 fusion proteins Key results: Effective therapeutic vaccine with histopathologic regression and significantly higher fold changes in their IFNγ	NCT02139267/ Completed/ [203]
II	Cervical cancer	Intramuscular injection and electroporation	Intervention: GX-188E DNA vaccine plus Pembrolizumab PD-1-blocking antibody Key results: This combination therapy showed preliminary antitumor activity	NCT03444376/ Active, not recruiting/ [204]
II	Cervical Cancer	Intramuscular injection	Intervention: VB10.16 vaccine (HPV16 E7/E6 protein linked to human chemokine MIP-1α) in combination with Atezolizumab PD-L1-blocking antibody Key results: No study results are available	NCT04405349/ Active, not recruiting/ [205]
II	Merkel Cell Carcinoma	Intratumural injection and electroporation	Intervention: DNA vaccine encoding IL-12 Key results: The vaccine is secure, and produces a systemic immune response, increased peripheral and intratumoral specific T cells	NCT01440816/ Completed/ [190]
II	Melanoma	Intratumural injection and electroporation	Intervention: DNA vaccine encoding IL-12 Key results: Circulating PD-1+ CD4+ and CD8+ T cells declined with treatment; specific immune responses to gp100 were also detected and were correlated with an increase in CD8^+^, CD3^+^ T cells within the tumor.	NCT01502293/ Completed/ [167]

## 10. RNA Vaccines

In recent years, RNA vaccines have gained substantial attention due to their rapid development and emergency approval for SARS-CoV-2 vaccination. RNA vaccines are similar to DNA vaccines (Table 3), as they are both easy to design, safe, and well tolerated in humans. Both are capable of eliciting humoral and cellular immune responses. Therefore, several RNA vaccines are under research for cancer treatment with promising results [206].

One of the principal disadvantages of RNA vaccines is their low stability, with the need to encapsulate the RNA molecules and low temperatures for storage and transport. Furthermore, after the use of mRNA COVID-19 vaccines in millions of people worldwide, some safety concerns have emerged and need to be addressed to improve this technology [207,208].

Notably, several DNA vaccines are being tested in humans for SARS-CoV-2 vaccination inducing durable humoral responses and the significant activation of CD8+ T cells with lytic potential, opening new opportunities for using DNA vaccines for viral prevention [209,210].

## 11. Future Perspectives

Over the past years, new and exciting knowledge about cancer cell biology and the immune system’s functions has emerged. This knowledge, in combination with new devices applicable for nucleic acid delivery and molecular biology tools for DNA manipulation, permits the design of novel strategies to fight cancer.

The development of more needle-free injection devices is a research area with great opportunity to improve the delivery of small amounts of drugs into the skin layers, such as in DNA vaccination [211,212].

One of the most attractive strategies in cancer treatment with promising results involves plasmid DNA for ex vivo modification of T cells, using transposons, designer nucleases, or CRISPR/Cas9 elements to target cancer cell recognition and elimination when returned to the patients [213,214]. However, ex vivo cell therapies are more expensive and require elaborate strategies.

Due to the variability in the intratumoral microenvironment and the diverse genetic profile of cancer cells between different patients (even with the same type of cancer), personalized treatments have emerged as a research area with increasing attention to generate an effective therapy capable of dealing with the disease in a more targeted way [215].

In clinical trials, the most effective interventions involve combined therapies, such as prime-boost strategies (where DNA vaccination is administered followed by the subsequent administration of other viral or non-viral vectors) or DNA vaccines combined with ICB therapy or other drugs. As mentioned before, these interventions are necessary to overcome the complexity of cancer.

Recently, other elegant strategies have been shown to induce a potent anti-tumor response in clinical trials. This finding is the case of the VB10.16 vaccine (Vaccibody). This vaccine consists of a plasmid that encodes a therapeutic protein composed of three elements; an E6/E7 antigen, a dimerization entity, and a MIP-1 α targeting unit that specifically binds to APCs. This vaccine has been demonstrated to induce potent immune responses in patients with HPV16+ cervical intraepithelial neoplasia (CIN) 2/3, eliciting CD8+ T cells and driving robust immune responses contributing to regression in lesion size (in 14 from 16 patients treated) and in lesion grade (CIN1/0) in eight patients [216]. The combination with ICB therapy is under investigation in a Phase IIa clinical trial to improve its antitumor effect [205].

Novel strategies, such as those discussed in this review, are under way to improve the use of DNA for in vivo gene therapy and vaccination. We expect that in the near future, they will receive approval for the prevention and treatment of cancer in humans.

## Figures and Tables

**Table 1:** Classification of tumor antigens.

Categories	Type of Antigen	Examples	References
Tumor-specific antigens	Viral antigens	L1, E6, and E7 from human papillomavirus (HPV)	[59,60]
		HBsAg from hepatitis B virus (HBV)	[61,62]
		Epstein–Barr nuclear antigens (EBNAs)	[63,64]
	Private neoantigens	Differs from each patient	[65]
	Public neoantigens	TP53	[66]
		KRAS	[67]
		PIK3CA	[68]
		Histone H3.3	[69]
Tumor-associated antigens	Overexpressed proteins	Receptor tyrosine-protein kinase erbB-2	[70,71]
		Epidermal growth factor receptor (EGFR)	[72]
		Mucin 1, cell surface associated (MUC1)	[73]
		Tumor protein D52 (TPD52)	[74]
		Mammaglobin A (Mam-A)	[75,76]
		Insulin-like growth factor (IGF) binding protein 2 (IGFBP-2)	[77]
	Differentiation antigens	Prostate-specific membrane antigen (PSMA)	[78,79]
		Prostatic acid phosphatase (PAP)	[80,81]
		Prostatic specific antigen (PSA)	[78,82]
		Carcinoembryonic antigen (CEA)	[83]
		Tyrosinase	[84]
		Glycoprotein 100 (gp100)	[85]
		Dickkopf-1 (DKK1)	[86]
	Cancer testis antigens	MAGE-A	[87,88]
		SSX-2	[89,90]
		NY-ESO-1	[91,92]

**Table 3:** Main advantages and disadvantages of non-viral vectors for cancer treatment.

	DNA	RNA
Advantages	Non-infective platforms	Non-infective platforms
	Easy to design and edit	Easy to design and edit
	Economic synthesis	Economic synthesis
	Induce specific immune responses	Induce specific immune responses
	High stability	Non-genetic integration
Disadvantages	Poor immunogenic	Poor immunogenic
	Low transfection efficiency	Low transfection efficiency
	Unknown side effects	Unwanted inflammatory responses
	May require a special administration device	Requires low temperatures for storage
	Potential integration into the human genome	Low stability

## Data Availability

Not applicable.
